# Chemoradiotherapy combined with NK cell transfer in a patient with recurrent and metastatic nasopharyngeal carcinoma inducing long-term tumor control: A case report

**DOI:** 10.1097/MD.0000000000022785

**Published:** 2020-10-23

**Authors:** Yuan-yuan Jin, Wen-zhuo Yang, Sen Zou, Zheng-yang Sun, Chun-tao Wu, Zhao-yong Yang

**Affiliations:** a NHC Key Laboratory of Biotechnology of Antibiotics, Institute of Medicinal Biotechnology, Chinese Academy of Medical Sciences, Beijing; b North China University of Science and Technology Affiliated Hospital, Tangshan, China; c Sun Yat-sen University School of Medicine, Guangzhou.

**Keywords:** case report, chemoradiotherapy, intracranial metastases, nasopharyngeal carcinoma, NK cell transfer

## Abstract

**Rationale:**

Nasopharyngeal carcinoma (NPC) is one of the most common malignancies in Southern China. Although combined chemotherapy with radiotherapy has been widely used in treating locally advanced lesions, relapse and metastases remain the primary cause of treatment failure, and are associated with an extremely poor prognosis. Therefore, more efficient and milder therapies are needed.

**Patient concerns:**

Herein, we report a patient with advanced NPC with intracranial metastases who showed progression during conventional treatment.

**Diagnoses:**

Nonkeratinizing undifferentiated nasopharyngeal carcinoma (stage IV).

**Interventions:**

After the completion of initial chemoradiotherapy and targeted therapy, metastases to brain occurred during follow-up. Ex vivo-cultured allogeneic NK cell infusion was offered.

**Outcomes:**

Although the intracranial metastases did not decrease 10 months after the NK cell treatment, they decreased significantly at 31 months after the treatment and partially disappeared. The tumor response indicated partial response. Furthermore, all of the intracranial metastases continued to decrease at about 42 months after treatment.

**Lessons:**

The brain metastases of NPC are rare with poor prognosis. Radiotherapy in NPC can disrupt the blood–brain barrier, which may contribute to the metastases of brain. This case report will provide rationale for NK cell infusion following regular chemoradiotherapy.

## 1 Introduction

Nasopharyngeal carcinoma (NPC), previously known as lymphoepithelioma, is one of the most common head and neck cancers, with endemic distribution in Southern China, Southeast Asia, Africa, and the Middle East.^[1]^ The rate varies from a minuscule value of <1 per 100,000 individuals in nonendemic areas to a high value of 25 to 30 and 15 to 20 males and females per 100,000 individuals in endemic areas, respectively.^[2]^ NPC can be categorized into 3 histological types: type I, keratinizing type (20%–25%); type II, nonkeratinizing differentiated type (10%–15%); type III, nonkeratinizing undifferentiated (60%–65%).^[3]^ The mainstay of treatment for NPC is radiotherapy in locoregional lesions as the nonkeratinizing variety is highly radiosensitive. Chemotherapy is preferred concomitantly with radiation in advance stages, whereas conventional chemotherapy and radiotherapy only have limited efficacy in NPC patients with late stage disease.

During recent decades, significant strides have been made in the fields of immunology and immunotherapy. Following the achievements of adjuvant immunotherapy using cytokine-induced killer (CIK) cells in the treatment of hepatocellular carcinoma (HCC), natural killer (NK) cell therapy has been discussed as a promising candidate for the next important advance. Since the discovery of NK cells, a great deal of research followed, which elucidated a critical role of NK cells in supporting the whole immune system, identified their association with many human diseases, and even attempted to use NK cells as a form of therapy.^[4]^ Of note, it was demonstrated in more recent studies that NK cells can identify and selectively kill cancer stem cells,^[5–7]^ suggesting that by targeting quiescent and nonproliferating cancer stem cells, NK cell-based therapy may become an effective method to prohibit relapse and metastasis.^[8]^

**Figure 1:**
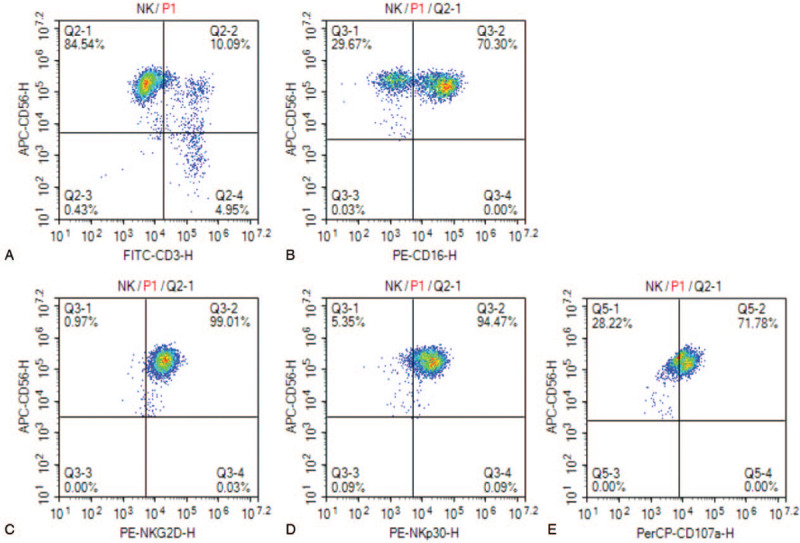
(A) Dot plots from one representative experiment depicting NK cell (CD3^-^CD56^+^) purity. (B) Dot plots from one representative experiment depicting NK cell (CD16^+^CD56^+^) purity. (C and D) Expression of activating receptors NKG2D and NKp30 on the expanded NK cells in the in vitro expansion protocol was evaluated by flow cytometry method. € The dot plots from one representative experiment illustrating CD107a surface expression on the NK cells after stimulation by the target cell line.

Until recent, 92% of clinical studies used NK cells from peripheral blood, either donor- (79% of recruiting trials) or patient-derived (13% of recruiting trials).^[9]^ Alternatives are the use of NK cell lines, or the differentiation of NK cells from umbilical cord blood (UCB) or pluripotent stem cells.^[10–12]^ Notably, UCB offers unique advantages, many of which are directly applicable to NK cell-directed alloreactivity. The ease of collection of UCB and cryopreservation makes them readily available as an off-the-shelf source for NK cell immunotherapy.^[13,14]^ Besides, the presence of almost a log fewer T-cells in UCB compared to other graft sources,^[15–19]^ most of which are naive,^[20–22]^ minimizes the risk of graft versus host disease.^[23–26]^ More importantly, NK cells reconstitute more rapidly after cord blood transplantation than peripheral blood haploidentical stem-cell transplantation.^[27,28]^ In view of this, we developed ex vivo expansion techniques that can induce cord blood mononuclear cells to directly differentiate into high cytotoxic NK (Fig. 1) using a cocktail of cytokines and interleukin-2. The culture method of high cytotoxic NK cells will be introduced in another article. Here, we report a case of NPC with recurrence and intracranial metastasis, who received our NK cell immunotherapy inducing long-term tumor control.

## 2 Case presentation

**Figure 2:**
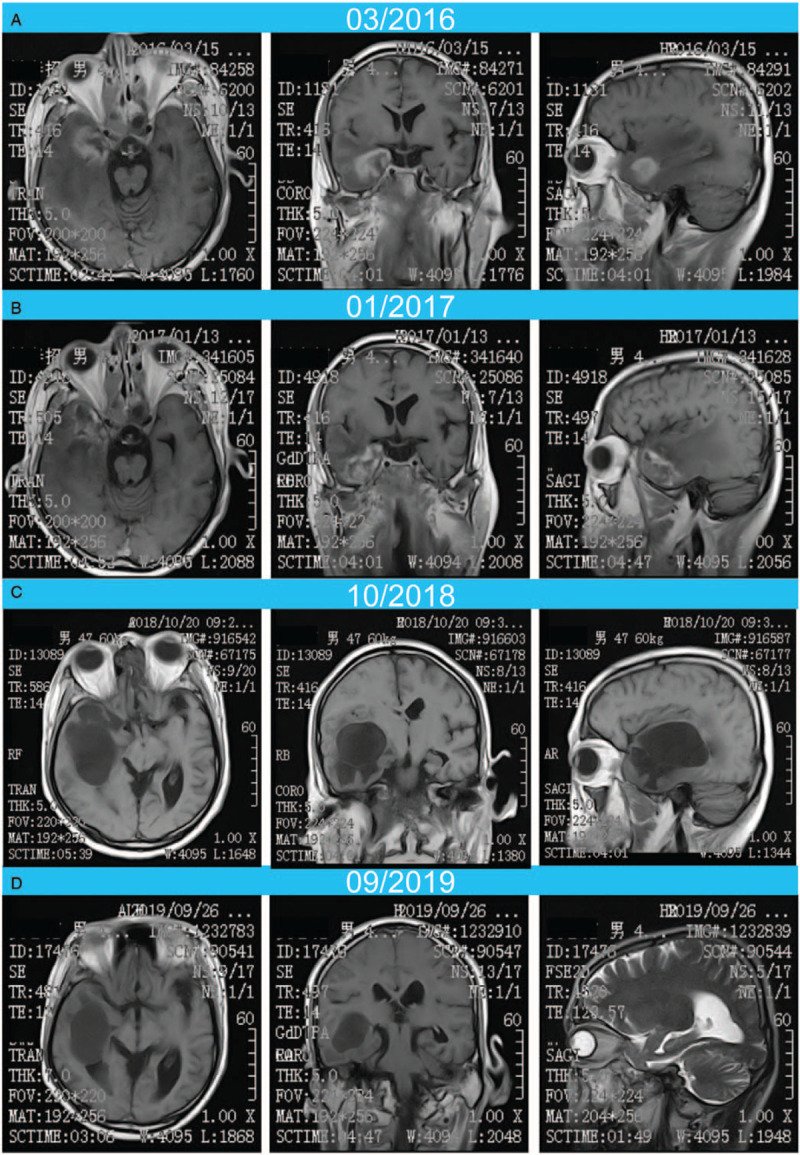
(A) The intracranial metastases before NK cell treatment (March, 2016); (B) the intracranial metastases 6 months after NK cell treatment (January, 2017); (C) the intracranial metastases about 2 years after NK cell treatment (February 26, 2018); (D) the intracranial metastases about 3 years after NK cell treatment (January 18, 2019).

**Figure 3:**
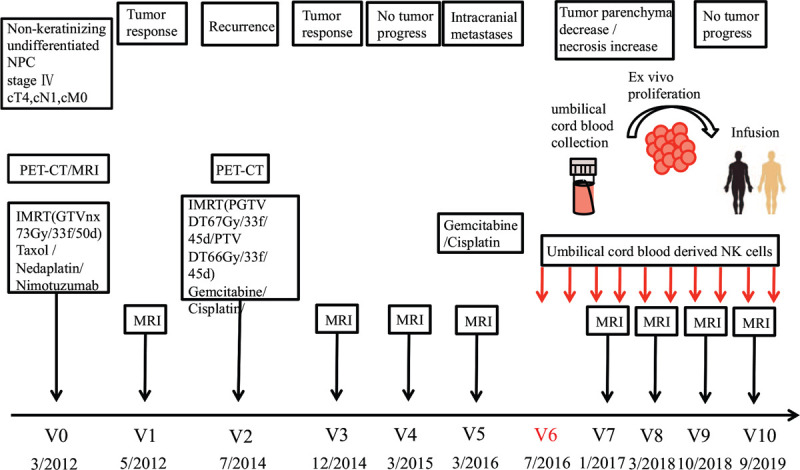
Schematic representation of the clinical history, therapy, and visits of a patient diagnosed with nonkeratinizing undifferentiated nasopharyngeal carcinoma (stage IV, cT4, cN1, cM0) in March, 2012 (V0). After concurrent RCT (paclitaxel /nedaplatin, 73Gy), a partial tumor response was evidenced by MRI scanning (V1). Two years later (V2, 7/2014), the patient received concurrent RCT (gemcitabine/cisplatinum, 67 Gy and 66 Gy), the state of the disease recurrence was evidenced much better than before by MRI scanning (V3). Three months later (V4), MRI scanning showed no tumor progression. On March, 2016, the MRI examination of brain indicated intracranial metastases (V5). On July, 2016 (V6), the patient began to receive NK cell treatment on a 3-yearly basis. MRI scanning after NK therapy (V7–V10) revealed that the intracranial metastases was gradually shrinking or even disappearing.

Written informed consent was obtained from the patient. A 48-year-old male was diagnosed with nonkeratinizing undifferentiated NPC with stage IV (cT4, cN1, cM0) based on the criteria of 8th AJCC/UICC edition in 3/2012. The patient was treated with concurrent paclitaxel/nedaplatin (TP)-based chemoradiotherapy (RCT) (03/2012–05/2012) with a total radiation dose of 73 Gy and the target therapy of nimotuzumab. Magnetic resonance imaging (MRI) examination after treatment (May 19, 2012) showed obvious reduction in tumor size. On July 21, 2014, the patient was readmitted to the hospital with a decreased vision of right eye and facial paralysis and diagnosed as recurrent NPC. Subsequently, the patient was treated with chemotherapy of gemcitabine/cisplatinum (GP) regimens and 2 cycles of intensity-modulated radiotherapy (IMRT) with a total radiation dose of 67 Gy and 66 Gy (July, 2014–September, 2014). After the completion of chemoradiotherapy, the tumor response indicated much better than before. For the next follow-up, there was no significant change between the MRI examination of nasopharynx on March, 2015 and December, 2014. Unfortunately, he was found to have intracranial metastases on March, 2016 by MRI scanning (Fig. 2). So the patient began to receive GP chemotherapy for 3 cycles and capecitabine afterwards to maintain chemotherapy due to intolerance. Then, NK cell treatment started on July, 2016, using ex vivo-generated NK cells from UCB, at a dose of 2 × 10^9^ CD56^+^/CD3^−^cells, intravenously, 3 times a year, up to now. Six months after NK therapy, MRI examination (January, 2017) showed nearly no change of the intracranial metastases (Fig. 2). Two years later, MRI examination (October, 2018) showed that all the intracranial metastases had begun to decrease significantly, and some metastases had disappeared (Fig. 2). Until the recent MRI examination on September, 2019, the intracranial metastases had continued to decrease (Fig. 2). At his last follow-up, about 3 years after initiating NK cell treatment, he was observed to be in a very good condition, without evidence of disease progression (V0, diagnosis; V1, MRI after RCT; V2, recurrence; V3-V4, MRI after RCT;V5, intracranial metastasis;V6, NK cell therapy after chemotherapy intolerance; V7–V10, MRI after NK cell therapy; Fig. 3).

## 3 Discussion

The rate of distant metastases occurrence is higher in locally advanced NPC, and the most common sites are the bone, lung, and liver.^[29]^ Central nervous system (CNS) metastasis of NPC is an extremely rare occurrence, although direct invasion to the skull base is not infrequent in patients at a locally advanced stage. Therefore, studies by far have failed to discuss its treatment and prognosis systematically, leaving only a few case reports.^[30,31]^ With the development of immune therapy, the treatment effects on NPC need to be urgently explored. In this report, we present 1 unique case of NPC with recurrence and metastasis to the right temporal lobe. At the same time, we also attempt to adopt NK cell therapy after the failure of conventional therapy.

Concurrent chemoradiotherapy with or without adjuvant chemotherapy, provides a benefit in overall survival and has become the standard treatment for locoregionally advanced NPC, although with acute toxicities.^[32,33]^ Nimotuzumab has marketing approval for the treatment of locoregionally advanced NPC,^[34,35]^ and the addition of induction chemotherapy to concurrent chemoradiotherapy and nimotuzumab could obtain the best survival benefits.^[36]^ The patient in our study received 2 cycles of chemotherapy consisted of paclitaxel and nedaplatin (TP) followed by radical IMRT combined with concurrent nimotuzumab after being diagnosed with advanced NPC and yielded promising objective response and local control. But unfortunately, there was local recurrence after 2 years. He then received concurrent chemoradiotherapy once again and had been sustained partial response by follow-up and imaging evaluation until March, 2016, intracranial metastases appeared. It has been reported that radiotherapy may destroy the blood–brain barrier in NPC patients.^[37]^ Qin et al^[38]^ reported in a retrospective study that irradiation with a 2-Gy-fraction dose resulted in maximal opening of the blood-brain barrier for over half a year. Moreover, Chan et al^[39]^ observed blood–brain barrier disruption by MRI in 89% of radiotherapy-treated, NPC patients, even 2 to 10 years after radiotherapy. Consistent with these reports, we reasoned that disruption of the blood–brain barrier might occur following radiotherapy in this patient, which resulted in intracranial metastases.

Chemotherapy with/without targeted therapy is the main treatment for NPC with distant metastasis, according to expert opinion.^[40]^ However, the treatment strategy for brain metastasis remains controversial. The prospect of these patients diagnosed with CNS was dismay. Most of patients in the reports suffered from exacerbated neurological deficits and succumbed finally.^[30,41]^ The patient in our study received a combined chemotherapy consisting of 3 cycles of gemcitabine and cisplatinum (GP). Due to intolerance to this chemotherapy regimen, GP was replaced with capecitabine to maintain chemotherapy.

Considering the uncertainty of chemotherapy and the intolerance, the patient turned to NK cell-based immunotherapy for a try. Previous studies exploring different allogeneic NK cell products showed promising antitumor activity in various cancers. However, for advanced NPC, this has not been clearly demonstrated yet. Here, we present the first study investigating our allogeneic UCB-NK cell product, which is highly activated and exhibits profound cytotoxic potential, in this NPC patient following intravenous infusion. In this case, we found that the brain metastases did not decrease at 6 months’ time, yet they decreased significantly about 2 years after treatment and some disappeared. Moreover, all of the brain metastases continued to decrease at about 3 years after treatment. Therefore, we think that NK cell therapy has late-onset and lasting antitumor effects in this patient. The toxicities were very mild, and the treatment was very well tolerated. At his last follow-up, he was observed to be in a very good condition.

## 4 Conclusion

In conclusion, our findings have interesting implications for current efforts to develop therapeutic strategies for NPC followed with recurrence and intracranial metastases and also suggest that NK cell therapy may be a promising option for the treatment of NPC after conventional radiotherapy and chemotherapy. However, caution should be paid to the possibility of the adverse effect of acute graft-versus-host disease for patients receiving allogeneic NK. The optimal dose of NK-cells and the follow-up treatment remain to be clarified. This is a report of one case; further well-designed and randomized studies with larger numbers of cases are needed to fully evaluate this strategy.

## Author contributions

The cell assay was done by Yuan-yuan Jin, Sen Zou and Zheng-yang Sun; Wen-zhuo Yang, Chun-tao Wu and Zhao-yong Yang conducted the clinical trial; The manuscript was prepared by Yuan-yuan Jin and Zhao-yong Yang.

All authors discussed the results and commented on the manuscript.
